# National Early Warning Score (NEWS) as an emergency department predictor of disease severity and 90-day survival in the acutely dyspneic patient – a prospective observational study

**DOI:** 10.1186/s13049-016-0273-9

**Published:** 2016-06-02

**Authors:** Bente Bilben, Linda Grandal, Signe Søvik

**Affiliations:** Institute of Clinical Medicine, Faculty of Medicine, University of Oslo, Oslo, Norway; Department of Anaesthesia and Intensive Care, Division of Surgery, Akershus University Hospital, 1478 Lørenskog, Norway

**Keywords:** Survival, Mortality, NEWS, MEWS, Emergency hospital service, Dyspnea, Chronic obstructive pulmonary disease

## Abstract

**Background:**

National Early Warning Score (NEWS) was designed to detect deteriorating patients in hospital wards, specifically those at increased risk of ICU admission, cardiac arrest, or death within 24 h. NEWS is not validated for use in Emergency Departments (ED), but emerging data suggest it may be useful. A criticism of NEWS is that patients with chronic poor oxygenation, e.g. severe chronic obstructive pulmonary disease (COPD), will have elevated NEWS also in the absence of acute deterioration, possibly reducing the predictive power of NEWS in this subgroup. We wanted to prospectively evaluate the usefulness of NEWS in unselected adult patients emergently presenting in a Norwegian ED with respiratory distress as main symptom.

**Methods:**

In respiratory distressed patients, NEWS was calculated on ED arrival, after 2–4 h, and the next day. Manchester Triage Scale (MTS) category, age, gender, comorbidity (ASA score), ICU-admission, ventilatory support, and discharge diagnoses were noted. Survival status was tracked for >90 days through the Population Registry. Data are medians (25–75th percentiles). Factors predicting 90-day survival were analysed with multiple logistic regression.

**Results:**

We included 246 patients; 71 years old (60–80), 89 % home-dwelling, 74 % ASA 3–4, 72 % MTS 1–2, 88 % admitted to hospital. NEWS on arrival was 5 (3–7). NEWS correlated closely with MTS category and maximum in-hospital level of care (ED, ward, high-dependency unit, ICU). Sixteen patients died in-hospital, 26 died after discharge within 90 days. Controlled for age, ASA score, and COPD, a higher NEWS on ED arrival predicted poorer 90-day survival. Increased NEWS also correlated with decreased 30-day- and in-hospital survival and a decreased probability for home-dwelling patients to be discharged directly home.

**Discussion:**

In respiratory distressed patients, NEWS on ED arrival correlated closely with triage category and need of ICU admission and predicted long-term out-of-hospital survival controlled for age, comorbidity, and COPD.

**Conclusions:**

NEWS should be explored in the ED setting to determine its role in clinical decision-making and in communication along the acute care chain.

## Background

A number of “track and trigger” or “early warning scores” (EWS) have been developed to detect patient deterioration [1–3]. *Aggregated* (multiple-parameter) and *weighted* (larger deviations give higher values) scores better predict unfavourable outcomes [4]. The National Early Warning Score (NEWS; Table 1) [3, 5] was developed and validated in hospital wards to detect patients with increased risk of unplanned ICU admission, cardiac arrest, and in-hospital death within 24 h. NEWS has not been validated in emergency departments (EDs), but studies of NEWS used in EDs are emerging [6, 7]. In this setting, the NEWS structure is recognisable from the “Airway – Breathing – Circulation – Disability – Exposure” viewpoint.

**Table 1:** National Early Warning Score (NEWS) value chart

	Score value	3	2	1	0	1	2	3
A B	Respiratory rate (breaths/min)	≤8		9–11	12–20		21–24	≥25
	O_2_ saturation (%)	≤91	92–93	94–95	≥96			
	Added O_2_		Yes		No			
C	Systolic BP (mmHg)	≤90	91–100	101–110	111–219			≥220
	Heart rate (bpm)	≤40		41–50	51–90	91–110	111–130	≥131
D	Level of consciousness				A			V, P, U
E	Temperature (C)	≤35.0		35.1–36.0	36.1–38.0	38.1–39.0	≥39.1	

*BP* blood pressure, *A* alert, *V* responds to verbal stimuli, *P* responds to pain only, *U* unresponsive to stimuli. In the NEWS scoring missing values are interpreted as normal values

The major difference between NEWS and previously used EWS is the inclusion of oxygen saturation (S_p_O_2_) in NEWS. Critics, fearing overtriage and alarm fatigue, have pointed to the technical difficulties of obtaining a representative S_p_O_2_ curve in peripherally vasoconstricted patients and to the chronically low S_p_O_2_ values found e.g. in patients with severe chronic obstructive pulmonary disease (COPD) [8]. Supporters of NEWS respond that the score was developed in general patient populations including many COPD patients, that peripheral vasoconstriction may be a severe circulatory sign, and that it is dangerous to a priori accept a poor S_p_O_2_ on the basis of a COPD diagnosis. Importantly, NEWS trigger thresholds may be individualised based on clinical judgement [5].

We wanted to evaluate how NEWS correlated with disease severity and hospital resource use in patients emergently presenting in the ED with respiratory distress. Many of these patients have chronic cardiorespiratory or neurological disease and chronically abnormal physiological readings. We found that NEWS on ED arrival in this patient subpopulation closely correlated with MTS triage category assigned by the ED nurse, ICU admission, and ventilatory support. NEWS predicted long-term, out-of-hospital survival, also when confounders such as age, a diagnosis of COPD, and other comorbidities were statistically adjusted for. We hope our findings spur further research on NEWS in the ED setting.

## Methods

This prospective, observational study took place at a general emergency hospital (708 somatic beds) serving a population of 493 000. In 2014, there were 62 200 somatic admissions to our institution, 77 % of which were non-elective. The hospital has a 10-bed general ICU, a 12-bed medical ICU, a cardiac high dependency unit (HDU), and a 21-bed combined postoperative unit/surgical HDU. As a rule, urgent and emergency care patients in Norway are evaluated at their general practitioner’s office or LEMC and triaged to hospital admission or home care. EDs are integrated divisions of the hospitals and receive mainly pre-triaged patients that are admitted to the hospital. In emergency situations, any person may call the Emergency Medical Communication Centre (EMCC) and acquire ambulance transport directly to a hospital.

### Patient inclusion

Study patients were a convenience sample included between Jul and Nov 2014. Eligible were patients >18 y emergently presenting in the ED with respiratory distress as a symptom, sign, or main complaint. Excluded were patients that were dead on arrival in the ED (no resuscitation attempted) or dead before any physiological measurements could be made. We attempted to include all eligible patients in the periods when the one of the recruiting researchers (medical students, BB and LG) were present. Recruitment periods were scheduled to obtain a representative sample of time slots with regard to day/evening/night shifts and day of week. We aimed to include 250 patients, because we decided that effects too small to be statistically detectable in a group of this size would be of little practical everyday importance.

### Ethical considerations

Patients in respiratory distress may be severely affected by poor oxygenation and respiratory work. Inclusion in this non-interventional study was therefore considered exempt from written patient consent by the Akershus University Hospital Data Protection Officer (Ref.no: 14-085), who in this matter acted on behalf of the Regional Committee for Medical and Health Research Ethics and the Norwegian Data Protection Authority. All included patients were explained the nature of the study and their right to withdraw if they wished as soon as they were deemed able to understand the information. Patients who never seemed able to comprehend this information, who died, or were transferred to a higher-level hospital before information could be given, were not excluded.

### Data sources and measurements

On arrival, all patients were routinely triaged by an ED nurse according to the Manchester Triage Scale (MTS) system [9]. Patient evaluation and physiological measurements necessary to calculate NEWS were obtained concurrently by the ED triage nurse or, as soon as possible, by the recruiting researcher. Measurements were repeated 2–4 h later and on the following day by the recruiting researcher, who also interviewed patients and reviewed patient records.

Physiological measurements were recorded as actual values and coded in a NEWS chart. Included variables were respiratory rate counted over 30 s, oxygen saturations (S_p_O_2_), body temperature (electronically recorded in the ear), systolic blood pressure, pulse rate counted over 30 s, and level of consciousness graded on the AVPU scale (Alert, Verbal = voice response present, Pain = pain response present, Unresponsive). Patients with a missing second or third NEWS scoring due to early discharge, transfer, unavailability due to e.g. imaging procedures, or death, were not excluded from analysis.

Comorbidities included in the Charlson Comorbidity Index were recorded on the basis of patient interviews and ICD-10 codes. Updated weights [10] were used. ASA-PS classification was defined as ASA 1: Healthy patient, ASA 2: Mild disease without systemic limitations, ASA 3: Moderate disease with defined systemic limitations, ASA 4: Severe disease that is a constant threat to life, 5: Moribund patient not expected to survive without surgery/intensive medical support. In this study, ASA score was used as a predictor and thus scored according to the patient’s status *prior to* the acute incident that had caused the ED visit.

Outcome measures were obtained by a second chart review of the hospital’s electronic patient system, a minimum of 90 days after ED arrival. We recorded length of hospital stay, maximum level of care (ICU, Medical ICU, Cardiac HDU, Ward, ED), use of mechanical ventilatory support, discharge destination, discharge diagnoses, and survival status up to this time point (automatically updated from the Norwegian Population Registry).

Prevalent discharge diagnoses were pooled into broad groups: Pneumonia/bronchitis [ICD-10 code groups J2, J15, J18, J69, A15], chronic obstructive pulmonary disease (COPD) [J42, J43, J44], asthma [J45, J46], pulmonary embolism [I26], pulmonary malignancy [C34, C78.0–3], cardiac failure [I50], atrial fibrillation [I48], sepsis [A40, A41], renal failure [N18], and anaphylactic reactions [T78, T88].

### Statistical analyses

Data were analysed in JMP Statistics 11.2.1 for Mac (SAS Institute, Medmenham Marlow, SL7 2 EB United Kingdom). Descriptive data were reported as median (25–75th percentile) unless otherwise stated. Two-group differences were analysed with the Wilcoxon test, Fisher’s Exact test, or the non-parametric Cochrane Armitage test for trend, as appropriate.

Logistic regression analysis was used to evaluate the effect of NEWS on ED arrival on 90-day, 30-day, and in-hospital survival. We wanted to statistically control for confounding variables, while considering the recommended minimum of ten events (in our case, deaths) per predictor evaluated in a multiple logistic regression model [11]. Candidate confounding predictors were age, COPD, and ASA score used to quantify overall burden of disease before the acute incident. The effect of possible predictors was first assessed univariably after grouping continuous variables. In the multiple regression, NEWS, ASA score and age were treated as linear co-variates, COPD as categorical (yes/no). The significance level was set to 0.05. Variables with a *p*-value of >0.10 were removed from the model.

An unadjusted 90-day survival plot was calculated for patients with NEWS on ED arrival <5 and ≥5, respectively. A NEWS value of ≥5 is the recommended trigger for an urgent clinical review [5].

## Results

We included 246 patients. One additional person was initially included but later wished to withdraw. Demographic, process mapping, and outcome variables are shown in Table 2. Almost 3/4 of these respiratory distressed patients were triaged as MTS 1–2. They were typically home-dwelling (89 %), and genders were equally represented. Median age was 71 years, but 10 % were older than 87 years. Many patients were active (24 %) or previous (41 %) tobacco smokers, and 74 % had significant comorbidities (ASA 3–4). Median length of hospital stay was 4 days, but 10 % of patients were admitted more than 15 days. Overall mortality was 6.5 % in-hospital, increasing to 17 % after 90 days. Two patients died in the ED shortly after arrival; 12 % were discharged directly from the ED.

**Table 2:** Demographic, process mapping, and outcome variables

Variables studied		Total N = 246
Female gender		128 (52)
Age (years)		70.5 (60–80)
Body Mass Index		25 (21–28)
ASA score^a^ *prior* to the acute illness	ASA 1	20 (8)
	ASA 2	44 (18)
	ASA 3	164 (67)
	ASA 4	18 (7)
Charlson Comorbidity Index^b^		2 (1–3)
Present tobacco smoker		59 (24)
Previous tobacco smoker		102 (41)
Never smoker		83 (35)
Present smokers: Cigarettes/day		15 (10–20)
Admitted from	Home	218 (89)
	Residential care home	12 (4)
	Nursing home	13 (5)
	Other hospital	3 (1)
Arrived by ambulance		141 (58)
Time of arrival		12 (10 am–2 pm)
ED triage level^c^	Critial Care Team	18 (8)
	Met by ED physician	21 (9)
	MTS 2	131 (55)
	MTS 3	53 (23)
	MTS 4	14 (6)
Time from arrival to	NEWS^d^ scoring 1	0:08 (0:03–0:20)
	NEWS scoring 2	2:45 (2:24–3:24)
	NEWS scoring 3	23:35 (20:40–25:43)
NEWS on 1st scoring	(*n* = 246)	5 (3–7)
NEWS on 2nd scoring	(*n* = 174)	5 (3–7)
NEWS on 3rd scoring	(*n* = 201)	5 (3–7)
Length of hospital stay (days)		4 (2–7)
Maximum level of care	ICU	4 (2)
	Medical ICU	19 (8)
	Cardiac HDU	15 (6)
	General ward	177 (72)
	ED	31 (13)
Mechanical ventilatory support	Intubated	4 (2)
	NIV or BiPAP	20 (8)
	CPAP	7 (3)
	None	216 (88)
Discharged to	Home	181 (74)
	Nursing home	38 (15)
	Rehabilitation facility	2 (1)
	Other hospital	9 (4)
	Dead	16 (7)
Discharged alive		230 (93)
Alive 30 d after ED arrival		224 (91)
Alive 90 d after ED arrival		204 (83)
Alive 90 d after ED arrival	MTS 1	25/39 (64)
	MTS ≥2	179/207 (86)
	Treated in ICUs or HDU	27/38 (71)
	Treated in ward or ED	177/208 (85)

Numbers are n (%) or median (25–75th percentile). Time is given as hours:minutes. ^a^ *ASA* American Society of Anesthesiologists Physical Status score prior to this acute incident. ^b^Charlson Comorbidity Index with updated weights. ^c^ *MTS* Manchester triage scale. ^d^ *NEWS* National Early Warning Score, *ICU* intensive care unit, *HDU* high dependency unit, *ED* emergency department, *NIV* non-invasive venitlation, *BiPAP* Bi-level positive airway pressure, *CPAP* continuous positive airway pressure

**Fig. 1:**
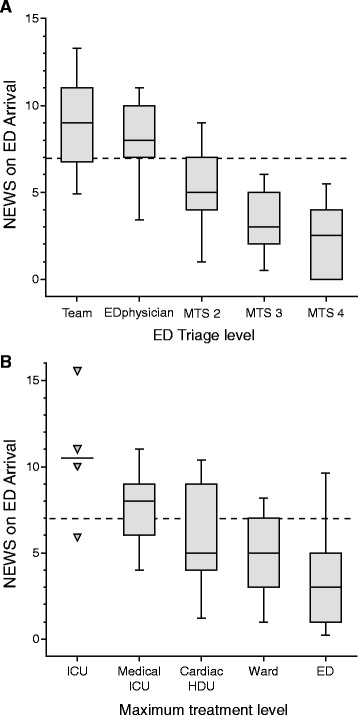
National Early Warning Score (NEWS) calculated on ED arrival versus **a** Manchester Triage Scale category (MTS) and **b** Maximum level of care during hospital stay, in 246 patients presenting with respiratory distress *Boxes* comprise 25th–75th percentiles with median value shown, whiskers display 10th and 90th percentiles

Median NEWS on ED arrival was 5, and 31 % of patients had a NEWS of ≥7. Of these, 30 % were treated in an ICU or HDU. Patients arriving by ambulance had higher NEWS than those arriving by private transportation (median 5.5 versus 4.0, Wilcoxon test *p* < 0.0001). NEWS on ED arrival scored by the recruiting researchers showed good correlation with the MTS category assigned by the triage nurse (Fig. 1a; Kruskal-Wallis Rank sums tests *p* < 0.001). For all MTS categories, 90-day survival decreased by increasing NEWS risk category (NEWS 0, 1–4, 5–6, ≥7; Cochrane Armitage trend test, *p* < 0.05 for all).

**Fig. 2:**
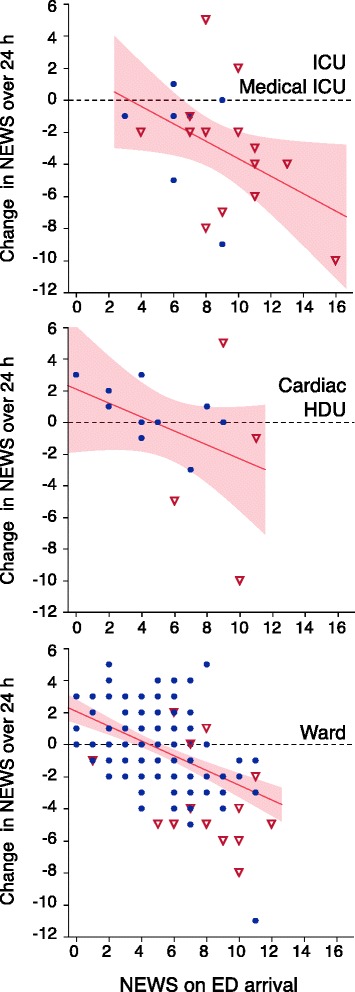
Change in NEWS from ED arrival to the following day versus initial NEWS, in 201 patients admitted with respiratory distress. Higher initial NEWS and larger reductions in NEWS were seen in patients admitted to ICUs (*upper panel*), and generally in patients triaged as MTS 1 (*open triangles*). Patients triaged as MTS ≥ 2 (*black dots*) and patients treated in general wards had lower initial NEWS but showed less improvement over the first 24 h

**Fig. 3:**
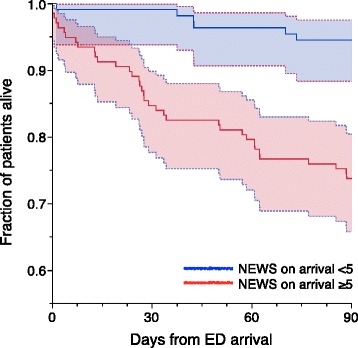
Survival plot of patients presenting in the ED with respiratory distress; 109 patients with NEWS <5 (*blue line*) on arrival and 137 patients with NEWS ≥5 (*red line*). Shaded areas display 95 % confidence areas. A higher NEWS value on ED arrival was associated with decreased long-term survival

Patients triaged to different levels of care differed regarding NEWS on ED arrival (Fig. 1b; Kruskal-Wallis *p* < 0.0001) and regarding improvement in NEWS from arrival to the following day (Fig. 2; Kruskal-Wallis *p* = 0.005). Accordingly, NEWS on arrival was higher for patients receiving mechanical ventilatory support (NEWS 7 (6–11)) versus those who did not (NEWS 5 (3–7)), and higher for home-dwelling patients that could *not* be discharged to their own home (NEWS 6.5 (5–8)) versus those who could (NEWS 4 (2–6)) (all: Wilcoxon test *p* < 0.0001). Patients with NEWS ≥5 on ED arrival had poorer 90-day survival than patients presenting with a NEWS of <5 (74 % versus 95 %; Fig. 3). MTS 1 patients and patients admitted to an ICU or HDU had poorer survival rates (Table 2).

Prevalent discharge diagnoses were pneumonia or bronchitis (22 % of patients), COPD (29 %), primary or secondary pulmonary neoplasm (8 %), asthma (4 %), pulmonary embolism (3 %), cardiac failure (24 %), atrial fibrillation (17 %), renal failure (4 %), anaphylactoid reaction (4 %), and sepsis (4 %). Descriptive diagnoses only (e.g. respiratory failure, dyspnea, chest pain) were found in 9 % of patients. Eighty-seven percent of patients had one or more of the above diagnoses.

Multivariable logistic regression analysis was performed only for 90-day survival (42 deaths in 246 patients), candidate predictors being NEWS on ED arrival, ASA score, age, and COPD (Tables 3 and 4). We found that an increase in NEWS on ED arrival, ASA score, and age all independently decreased 90-day survival. Simultaneously, for a given NEWS, ASA score, and age, a patient with COPD had higher predicted 90-day survival than one with other discharge diagnoses (Table 4). Univariable logistic regression analyses showed that a higher NEWS on ED arrival also correlated with poorer in-hospital and 30-day survival (16 and 23 deaths in 246 patients, respectively; Table 4).

**Table 3:** Crude 90-day survival rates by various predictors

Predictor	Category	N	Patients alive 90 days after arrival n (%)
NEWS on ED arrival*	0	13	13 (100)
	1–4	96	90 (94)
	5–6	61	49 (80)
	≥7	76	52 (68)
ASA score*	1	20	20 (100)
	2	44	42 (96)
	3	164	133 (81)
	4	18	9 (50)
Age*	<40	18	18 (100)
	40–49	16	15 (94)
	50–59	31	27 (87)
	60–69	58	52 (90)
	70–79	66	54 (82)
	80–89	44	31 (70)
	≥90	13	7 (54)
COPD^a^	No	175	142 (81)
	Yes	71	62 (87)

*NEWS* National Early Warning Score, grouped by risk categories [5]. *ASA score* American Society of Anesthesiologists Physical Status score prior to this acute incident

*Cochrane Armitage trend test *p* < 0.0001. ^a^Fishers Exact test: *p* = 0.28

**Table 4:** Results from logistic regression analyses

	Term	Chi square	*p* value	Unit OR	95 % CI
Alive at 90 days	Intercept	30.33	<0.0001		
AUROC = 0.809	NEWS on arrival	6.72	0.0095	0.835	(0.725–0.954)
	ASA score	13.34	0.0003	0.188	(0.072–0.438)
	Age	8.29	0.0040	0.952	(0.918–0.982)
	COPD	6.09	0.0136	3.189	(1.313–8.120)
Alive at 30 days	Intercept	46.28	<0.0001		
	NEWS on arrival	12.23	0.0005	0.770	(0.661–0.888)
Discharged alive	Intercept	42.41	<0.0001		
	NEWS on arrival	11.80	0.0006	0.743	(0.621–0.875)

Only 90-day survival was multivariably evaluated. Linear covariates: NEWS: National Early Warning Score. ASA score: American Society of Anesthesiologists Physical Status score prior to this acute incident. Age measured in years. Categorical covariate: *COPD* chronic obstructive pulmonary disease, *AUROC* area under receiver operating curve. Unit OR: For each unit change in the predictor, the odds for being alive at the given time point improves by this factor

## Discussion

In this observational study of patients emergently presenting in the ED with respiratory distress, National Early Warning Score (NEWS) on arrival correlated closely with the assigned Manchester Triage Scale (MTS) category, need of treatment in a HDU or ICU, need of mechanical ventilatory support, discharge to one’s own home, and short- and long-term out-of-hospital survival. NEWS was an independent predictor of 90-day survival, controlled for patient age, COPD, and ASA category prior to the acute incident.

At the time of study, NEWS was not yet introduced in our ED. Scoring was performed by two medical students (BB and LG); treating doctors and nurses were unaware of the details of the scoring system and the calculated NEWS values. Thus we were able to compare MTS and NEWS prospectively in a “naive” setting.

### NEWS as a supplement in ED triage

NEWS is an aggregated and weighted summation of patient variables routinely observed and acted upon. Accordingly, NEWS on ED arrival correlated well with clinical choices that are based on overall patient evaluation, e.g. whether the EMCC decided to dispatch an ambulance, or whether prehospital services requested a critical care team. The MTS category assigned by the triage nurse also correlated well with the concurrently calculated NEWS (Fig. 1a). These findings indicate that information on patient status conveyed from the ambulance service shortly before patient arrival could allow EDs to calculate an expected NEWS value and to better tailor their response to emergency admissions.

No studies have yet established NEWS thresholds to trigger clinical actions in the ED. In our material, 3/4 of MTS 1 patients and 1/4 of MTS 2 patients had NEWS ≥7 on arrival (Fig. 1). On a hospital ward, this is the trigger for a critical care team evaluation [5]. Possibly, a combination of the NEWS and MTS systems could improve ED triage.

### NEWS on ED arrival and ICU admission

NEWS was designed for hospital wards, to detect patients with increased risk of death or unplanned ICU admission [3], but comparable predictive effects in ED patients have been demonstrated in general medical [6] and septic [7] populations. Our study extends these findings to acutely dyspneic patients presenting in the ED. The distribution of NEWS values among ICU admitted patients in our material was similar to that of ICU admitted septic patients [7]. However, average NEWS values in ICU patients will vary depending on the definition, availability, and use of ICU and HDU beds in a health system.

Patients received by critical care teams or immediate ED physician evaluation (MTS 1) and patients admitted to ICUs were more physiologically deranged on arrival but also showed largest NEWS improvement (Fig. 2). In contrast, MTS ≥2 patients treated in regular wards overall showed no change in NEWS from ED arrival to the following day, while their survival rate was far better than that of the ICU groups (Table 2). A simple numerical relation between change in NEWS and prognosis cannot be expected.

### NEWS and long-term out-of-hospital survival

Hospitals differ regarding whether critically ill patients remain admitted, are transferred to higher-level treatment, or are discharged to palliative care. Consequently, comparison of crude in-hospital survival rates is unreliable. Previous studies found that NEWS predicted short-term (24 h [3] or 48 h [6]) and in-hospital survival rates up to 30 days [6, 7]. We tracked every patient’s out-of-hospital survival status and demonstrated that NEWS on ED arrival predicted 90-day survival, also after adjusting for age, ASA score, and COPD (Table 4, Fig. 3). This is analogous to findings in Finnish hospital ward patients [4].

Many patients in our study were elderly, with a high burden of disease. Overall mortality was considerable (Fig. 3), and 62 % of deaths occurred after discharge. The need for out-of-hospital survival data in scientific studies is obvious. Decisions on treatment restrictions were not recorded or evaluated in our study.

### Other determinants of treatment and outcome

A multitude of factors determine treatment approaches and patient outcomes. Though NEWS was independently associated with survival (Table 4) and differed clearly between MTS groups and levels of care (Fig. 1), there were overlaps in NEWS between groups that our data cannot explain. The MTS system itself is supported by limited scientific documentation [12, 13]. Age and ASA score did not differ between MTS groups or levels of care for the 88 % of patients that were not directly discharged from the ED (data not shown). The expected reversibility of the clinical condition was a probably a key factor in deciding treatment.

Chronic hypoxemia affects baseline NEWS values. To prevent unwarranted oxygen therapy and unnecessary team calls to hospital wards, an alternative EWS has been proposed for patients with chronically low S_p_O_2_ [8]. In our study, 29 % of included patients had COPD as a discharge diagnosis, and the association between NEWS and survival was independent of COPD. Still, a given NEWS in a COPD patient predicted a lower reduction in survival than the same NEWS in a non-COPD patient. Among patients with ED, ward, or HDU as maximum level of care, those with COPD had significantly higher NEWS than non-COPD patients (data not shown). Interestingly, this was not so in patients treated in ICUs, maybe as a result of stricter patient selection.

### NEWS in communication between hospital and community heath services

NEWS and other early warning scores are being introduced in Norwegian community health services. This opens possibilities of a “common language” that may improve communication along the acute care chain – e.g. between a nursing home, EMCC, prehospital service, and ED. In contrast, reports of in-hospital NEWS values in patient discharge notes would probably only be useful for community doctors and nurses if accompanied by an evaluation, since a high NEWS may have been caused by a fully reversed condition, e.g. an episode of critical bleeding, or by incurable disease.

### Strengths and limitations

Our findings should be generalisable to populations of respiratory distressed patients emergently admitted to general acute-care hospitals. In institutions treating more selected patient groups, associations between NEWS and outcome could be different.

Health system organisation strongly influences ED population characteristics and outcomes, reducing the external validity of studies of ED scoring systems [12]. In our material of respiratory distressed ED patients, 88 % were admitted to the hospital and 72 % were MTS 1–2. Conversely, in a large study of MTS and survival in general ED patients the corresponding values were 10 and 26 % [14]. Clearly, comparison of results from differently organised systems must be done with caution.

A limitation of our study is its small size. Data was retrieved by two trained and dedicated students, this improved consistency and follow-up rates. A strength of our study compared to previous work is the completeness of our mortality data and the multivariable approach controlling for simultaneous patient risk factors.

## Conclusion

In patients emergently presenting with respiratory distress, NEWS on ED arrival correlated closely with MTS category and in-hospital treatment intensity. The relation between NEWS and 90-day survival was statistically independent of patient age, a COPD diagnosis, and ASA category prior to the acute incident. NEWS should be further explored in ED settings, to determine its role in clinical decision-making and communication along the acute care chain.

## Abbreviations

ASA, American Society of Anesthesiologists’ Physical Status Classification System; BMI, body mass index; COPD, chronich obstructive pulmonary disease; ED, emergency department; EMCC, emergency medical communication centre; EMS, emergency medical service; HDU, high dependency unit; ICU, intensive care unit; LEMC, local emergency medical centre; MTS, Manchester triage scale system; NEWS, National Early Warning Score
